# A 346 Case Analysis for Laparoscopic Spleen-Preserving No.10 Lymph Node Dissection for Proximal Gastric Cancer: A Single Center Study

**DOI:** 10.1371/journal.pone.0108480

**Published:** 2014-09-29

**Authors:** Chang-Ming Huang, Jun-Rong Zhang, Chao-Hui Zheng, Ping Li, Jian-Wei Xie, Jia-Bin Wang, Jian-Xian Lin, Jun Lu, Qi-Yue Chen

**Affiliations:** Department of Gastric Surgery, Fujian Medical University Union Hospital, Fuzhou, Fujian Province, China; Taipei Medical University, Taiwan

## Abstract

**Purpose:**

This study was designed to formulate a model that efficiently predicts splenic hilar lymph node metastasis (SHLNM) in patients with proximal gastric cancer and to assess indications for laparoscopic spleen-preserving no.10 lymph node dissection (LSPNo.10LND) based on this model.

**Methods:**

Patients (N = 346) with proximal gastric cancer who underwent LSPNo.10LND from January 2010 to October 2013 were prospectively enrolled and retrospectively evaluated. Groups of patients with and without SHLNM were compared, and independent risk factors for SHLNM determined. An optimal predictive model of SHLNM in patients with proximal gastric cancer was well established.

**Results:**

Of the 346 patients with proximal gastric cancer, only 35 (10.1%) were diagnosed with SHLNM. Depth of invasion, tumor location and metastases to No.7 and No.11 lymph nodes (LNs) were independent risk factors for SHLNM (p<0.0001 each). A model involving depth of invasion, tumor location and metastasis to No.7 and 11 LNs yielded a lowest Akaike’s information criterion (AIC) of −913.535 and a highest area under the ROC curve (AUC) of 0.897(95%CI:0.851–0.944). Stratification analysis showed no SHLNMs in the absence of serosal invasion of the lesser curvature and metastases at No.7 and No.11 LNs (T2-3∶0/87, 95% CI: 0.00–4.15).

**Conclusions:**

A model including depth of invasion, tumor location and metastases at No.7 and No.11 LNs was found optimal for predicting SHLNM for proximal gastric cancers. LSPNo.10LND may be avoided when tumors on the lesser curvature did not show serosal invasion or metastases at No.7 and No.11 LNs.

## Introduction

Proximal gastric cancer is defined as a tumor located in the upper 1/3 of the stomach [1]. According to JCGC 3^rd^ edition guideline [2], for advanced proximal gastric cancer, no.10 lymph node dissection (LND) should be involved in D2 lymphadenectomy with total or proximal gastrectomy. Since spleen-preserving no.10 LND (SPNo.10LND) does not significantly affect patient survival, morbidity and mortality rates when compared with no.10 LND plus splenectomy [3]–[8], the former is increasingly adopted in patients with proximal gastric cancer. Obstacles to SPNo.10LND include deep location, splenic vascular variation, narrow anatomical space, proneness to capsular tearing and complicated adjacent structures. En bloc resection requires the spleen to be mobilized outside the abdominal cavity, along with the body and tail of the pancreas [9]. This procedure is not only technically difficult, but can increase postoperative complications such as spleen reversal, preventing SPNo.10LND from being a routine method. Laparoscopic methods can better delineate the perigastric fascia, intrafascial space, vasculature and other structures, enhancing the safety of lymphadenectomy in the splenic hilar area. However, only few centers could independently complete laparoscopic SPNo.10LND (LSPNo.10LND) [10]–[14]. To date, LSPNo.10LND remains controversial for all advanced proximal gastric cancer, and there is no well-established predictive model for splenic hilar lymph node metastasis (SHLNM). This study was designed to formulate the optimal model for SHLNM and explore indications for LSPNo.10LND.

## Methods

### Patients

The 346 patients involved in this study underwent LSPNo.10LND at the Department of Gastric Surgery of the Fujian Medical University Union Hospital from January 2010 to October 2013. Patient characteristics were recorded prospectively in our clinical database and used for a retrospective analysis in this study.

Patients were included if they 1) were preoperatively diagnosed with proximal gastric cancer by gastroscopic biospy; 2) had no distal metastasis on abdominal and pelvic computed tomography (CT) or on abdominal ultrasound, as well as no evidence of lesions in the perigastric organs and LNs around the abdominal aorta; and 3) had undergone D2 lymphadenectomy with pathological R0 resection.

Patients were excluded if 1) they had proximal gastric cancer intraoperatively diagnosed as T4b tumors; 2) distant metastases were detected before or during the operation; 3) they had preoperatively received neoadjuvant chemo- or radiotherapy; 4) detailed clinical information was lacking; or 5) they had a history of previous gastrectomy.

Of the 346 patients with proximal gastric cancer, 35 had SHLNM (metastasis group) and 311 did not (non-metastasis group). The 346 patients consisted of 267 males and 79 females, of mean age 61.4±10.6 years and with a mean tumor size of 54.74+25.25 mm. Forty-five patients (13.0%) were classified as stage I, 83 (24.0%) as stage II and 218 (63.0%) as stage III [15]. Tumor diameters were assessed as being located in the lesser curvature, greater curvature, anterior wall and posterior wall according to JCGC 3^rd^ edition guideline [2].

### Surgical Procedures

Depending on the tumor location, patients underwent laparoscopic assisted total gastrectomy (LATG) or laparoscopic assisted proximal gastrectomy (LAPG) with standard D2 lymphadenectomy. In brief, the procedure of LSPNo.10LND was performed as follow:

The patient is placed in the reverse Trendelenburg position with the head elevated approximately 15 to 20 degrees and tilted left side up at approximately 20 to 30 degrees. The surgeon stands between the patient’s legs, with the assistant and camera operator both on the patient’s right side.

First step-dissection of lymph nodes in the inferior pole region of the spleen: The assistant places the free omentum in the anterior gastric wall and uses his or her left hand to pull the gastrosplenicligament. The surgeon gently presses the tail of the pancreas and separates the greater omentum toward the splenic flexure of the colon along the superior border of the transverse mesocolon. Next, the anterior pancreatic fascia (APF) is peeled toward the superior border of the pancreatic tail, along the direction of the pancreas. Then, the peeled anterior lobe of the transverse mesocolon (ATM) and APF are completely lifted toward the cephalad, to expose fully the superior border of the pancreas and enter the retropancreatic space (RPS). The lower lobar vessels of the spleen (LLVSs) or lower pole vessels of the spleen can then be exposed. The assistant’s right hand pulls up the lymphatic fatty tissue on the surface of the vessels, and the surgeon uses the non-functional face of the ultrasonic scalpel to dissect these lymphatic tissues, closing toward the vessels. The left gastroepiploic vessels (LGEVs) can then be revealed. Next, the assistant gently pulls the LGEVs, while the surgeon meticulously separates the fatty lymphatic tissue around them to denude them completely, then dividing them at their roots with vascular clamps. The division point is used as the starting point for the splenic hilar lymphadenectomy, to skeletonize one or two branches of the short gastric vessels (SGVs), which are divided at their roots toward the direction of the splenic hilum.

Second step-dissection of the lymph nodes in the region of the splenic artery trunk: The assistant places the free omentum between the inferior border of the liver and the anterior gastric wall and continually pulls the greater curvature of the fundus to the upper right, while the surgeon’s left hand presses the body of the pancreas. The assistant’s right hand pulls the isolated fatty lymphatic tissue on the surface of the splenic artery trunk. The surgeon denudes the middle of the splenic artery trunk until the crotch of the splenic lobar arteries lies along the latent anatomic spaces on the surface of the splenic vessels. The posterior gastric artery, which derives from the splenic artery, will always be encountered in this region; at this time, the assistant should clamp and pull the vessels upward, while surgeon denudes them and closes toward the splenic artery trunk. Then, the surgeon divides them at their roots with vascular clamps and completely dissected the fatty lymphatic tissue around the splenic vessels (No.11d).

Third step- dissection of lymph nodes in the superior pole region of the spleen: The assistant continually pulls the greater curvature of the fundus to the lower right, while the surgeon’s left hand presses the vessels of the splenic hilum. Then, taking the division point of the LGEVs as the starting point, the assistant gently pulls up the fatty lymphatic tissue at the surface of the terminal branches of the splenic vessels and keeps it under tension, while the surgeon uses the non-functional face of the ultrasonic scalpel to cut the surface of the terminal branches of the splenic vessels, completely skeletonizing the vessels in the splenic hilum with meticulous sharp or blunt dissection. During the dissection process, two or three branches of the SGVs arise from the terminal branches of the splenic vessels and enter the GSL. At this time, the assistant should clamp and pull the vessels upward, while the surgeon meticulously dissects the surrounding fatty lymphatic tissue, closing toward the roots of the SGVs. Next, the surgeon divides the vessels at their roots with vascular clamps after confirming their destinations in the wall of the stomach. In particular, the last SGV in the superior pole region of the spleen is often very short and easy to damage, causing bleeding. At this time, the assistant should adequately pull the fundus to the lower right to expose the vessel completely and should assist in the careful separation of the surgeon. Then, the separation is continued to dissect completely the fatty lymphatic tissue in front of the splenic hilar. Dissection of the lymph nodes behind the splenic vessels can be performed when the tail of pancreas is located in the inferior border of the spleen, and it lies a certain distance from the splenic hilum. The assistant’s left hand then ventrally lifts the termini of the splenic vessels using atraumatic grasping forceps, and the surgeon’s left hand presses the Gerota’s fascia. Then, the ultrasonic scalpel dissects the adipose tissue behind the splenic vessels in front of Gerota’s fascia. Attention is required during this step so that the separation plane does not exceed Gerota’s fascia, because doing so can damage the kidney, adrenal gland and related vessels or the nerves behind them. At this point, the splenic hilar lymphadenectomy is complete. An intraoperative view after splenic hilar lymphadenectomy is shown after the procedure. This procedure is what we call Huang's three-step maneuver for LSPNo.10LND. The results of this surgical technique are shown in Figure S1, Figure S2.

### Ethics Statement

Ethics committee of Fujian medical union hospital approved this retrospective study. Written consent was given by the patients for their information to be stored in the hospital database and used for research.

### Statistical analysis

All the statistical analysis and graphics were performed with the SPSS 20.0 and the R project. Univariate analysis was operated to determine the risk factors. Binary Logistic Regression confirmed the independent risk factors. Akaike’s information criterion (AIC) and the area under the ROC curve (AUC) were simultaneously evaluated the optimal predictive model of SHLNM. Through Stratification analysis and binary distribution test, we advocate the indication for LSPNo.10LND. All p value was lower than 0.05.

## Results

### 1. Univariate analysis of risk factors for SHLNM

The metastasis group consisted of 35 patients (10.1%) and the non-metastasis group of 311 patients (89.9%). None of early proximal gastric cancer in the metastasis group, compared with 35 of 311 (11.3%) had a advanced proximal gastric cancer s in the metastasis group. The average number of splenic hilar lymph nodes (SPHLNs) harvested was 2.69±2.76, 4.03±2.49 in the metastasis group and 2.56±2.80 in the non-metastasis group. Table 1 show the clinicopathological features of the various subgroups. SHLNM was significantly associated with tumor location (p<0.001), tumor size (p<0.001), CA-199 status, depth of invasion (p<0.001), UICC N classification, and lymph node metastasis (LNM) at No.1–4, No.6–9, No.11 and No.12 LNs (p<0.05 each), but not at No.5 LNs.

**Table 1:** Clinicopathologic features of patients with and without SHLNM.

	SPHLN			
Parameter	All patients (n = 346)	Non-metastasis (n = 311)	Metastasis (n = 35)	p value
Age, yr				
≤60	155	137	18	0.405
>60	191	174	17	
Sex				
Male	267	239	28	0.674
Female	79	72	7	
BMI, kg/m^2^				
<25	302	273	29	0.407
≥25	44	38	6	
Histology				
undifferentiated	198	175	23	0.529
mix	66	60	6	
differentiated	82	76	6	
Location				
Lesser	256	246	10	<0.001
Greater	22	18	4	
Anterior	5	3	2	
Posterior	17	12	5	
Circum	46	32	14	
Size (cm)				
<5.5	180	174	6	<0.001
≥5.5	166	137	29	
LVI				
LVI(−)	186	172	14	0.540
LVI(+)	160	139	21	
CA199(U/mL)				
≤37	293	268	25	0.043
>37	53	43	10	
Surgery				
LAPG	4	4	0	0.500
LATG	342	307	35	
UICC(n)				
IA	27	27	0	<0.001
IB	18	18	0	
IIA	43	43	0	
IIB	40	40	0	
IIIA	47	45	2	
IIIB	71	66	5	
IIIC	100	72	28	
Depth				
pT1	35	35	0	<0.001
pT2	26	26	0	
pT3	132	128	4	
pT4	153	122	31	
LNM				
pN0	87	87	0	<0.001
pN1	51	50	1	
pN2	61	56	5	
pN3	147	118	29	
Number of SPHLNs	2.69±2.76	2.56±±2.80	4.03±2.49	0.003
Number of dissection	41.44±15.45	41.55±15.81	40.40±11.93	0.676
No.1	76(346)	63(311)	13(35)	0.022
No.2	79(346)	61(311)	18(35)	<0.001
No.3	235(346)	202(311)	33(35)	<0.001
No.4	81(346)	60(311)	21(35)	<0.001
No.5	30(346)	24(311)	6(35)	0.074
No.6	48(346)	38(311)	10(35)	0.008
No.7	140(346)	112(311)	28(35)	0.001
No.8	54(346)	42(311)	12(35)	0.001
No.9	86(346)	69(311)	17(35)	0.001
No.11	60(346)	42(311)	18(35)	<0.001
No.12	43(346)	34(311)	9(35)	0.012

Circum, includes more than 2 portions and circum patients; BMI, body mass index; LVI, lymphovascular invasion; LNM, lymph nodes metastasis.

No.1(Right cardial), No.2(Left cardial), No.3(LN along the lesser curvature ), No.4(LN along the greater curvature), No.5(Suprapyloric), No.6 (Infrapyloric), No.7(LN along the left gastric artery), No.8(LN along the common hepatic artery), No.9(LN along the celiac artery), No.11(LN along the splenic artery ), No.12(LN in the hepatoduodenal ligament ).

### 2. Multivariate analysis of risk factors for SHLNM

A binary logistic regression model evaluating 15 factors found that four were independent risk factors for SHLNM: depth of invasion, tumor location, metastasis status at No.7 LNs, and metastasis status at No.11 LNs (Table 2). Compared with tumors located in the lesser curvature, those located in the greater curvature (OR 8.630), anterior wall (OR 42.265), posterior wall (OR 15.949), and circum (OR 9.678) had significantly higher risks of SHLNM (p<0.05 each).

**Table 2:** Binary logistic regression analysis of independent risk factors for SHLNM.

Variable		OR	95%CI		p value
			Lower	Upper	
Depth					
	T4a vs T2–3	5.921	1.786	19.628	0.004
Location					0.001
	Greater vs Lesser	8.630	1.942	38.352	0.005
	Anterior vs Lesser	42.265	2.516	710.016	0.009
	Posterior vs Lesser	15.949	3.583	71.003	0.001
	Circum vs Lesser	9.678	3.395	27.593	0.001
No.7	positive vs negative	3.996	1.429	11.176	0.008
No.11	positive vs negative	4.513	1.725	11.801	0.002

### 3. Comparisons of predictive models

**Figure 1:**
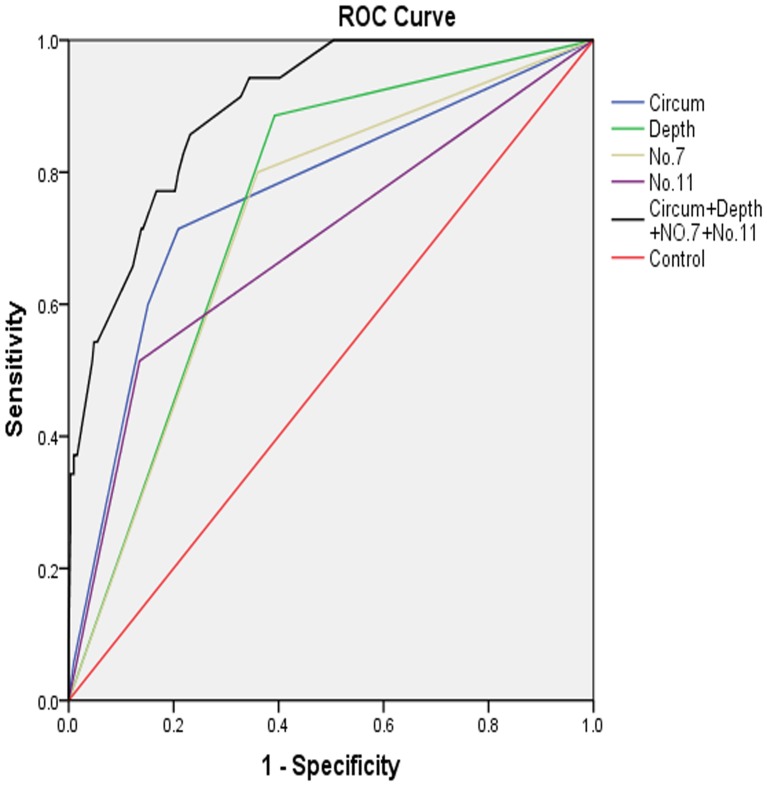
ROC curves assessing the accuracy of various predictive models.

To determine the optimal model for SHLNM, the area under the ROC curve (AUC) and the Akaike information criterion (AIC) were applied to various predictive models. The model that included tumor location, depth of invasion, and metastasis statuses at No.7 and No.11 LNs showed the highest accuracy (AUC 0.897, 95% CI0.851–0.944) and the lowest AIC score (−913.535; Table 3, Figure 1).

**Table 3:** Comparison of different predictive models for SHLNM.

Regression model	Lymph node status		AIC	AUC	95%CI(lower-upper)
	No.7	No.11			
Depth	−	−	−858.154	0.747	0.673–0.821
	+	−	−869.669	0.816	0.760–0.872
	−	+	−876.880	0.811	0.744–0.879
	+	+	−881.525	0.845	0.792–0.898
Location	−	−	−869.265	0.763	0.671–0.855
	+	−	−890.473	0.842	0.766–0.918
	−	+	−901.094	0.839	0.766–0.913
	+	+	−909.216	0.868	0.800–0.936
Depth+Location	−	−	−886.955	0.844	0.781–0.906
	+	−	−898.971	0.883	0.830–0.935
	−	+	−908.990	0.884	0.833–0.936
	+	+	−913.535	0.897	0.851–0.944
No.7	+	−	−851.856	0.720	0.636–0.804
No.11	−	+	−858.710	0.690	0.585–0.794
No.7+No.11	+	+	−869.092	0.775	0.689–0.861

### 4. Stratification analysis for SHLNM

Because the incidence of SHLNM was much lower for tumors located in the lesser curvature than for the other four locations, the latter four were combined. None of the advanced proximal gastric cancer in the lesser curvature, without serosal invasion and without metastases at No.7 and No.11 LNs, were found to have SHLNM, as determined by stratification analysis and binary distribution tests based on the four independent risk factors of SHLNM (T2-3∶0/87, 95%CI 0.00–4.15). Accuracy analysis of confidence intervals (CI) for a binary distribution showed that, when the number of samples was close to 100, the smallest 95%CI ranged from 0.00 to 8.30, smaller than those observed previously, indicating an extremely low risk for SHLNM. In contrast, patients with tumors located in other portions of the stomach, without serosal invasion and without metastases at No.7 and No.11 LNs had a larger 95% CI, indicating that they had somewhat risk of SHLNM (n = 10, p = 0.05, L = 16) [16]. Moreover, changes in any of these factors enhanced the risks of SHLNM (Table 4).

**Table 4:** Stratification analysis of SHLNM for advanced proximal gastric cancer.

Location	No.7 & No.11	Depth	Ratio(%)	95%CI	Shortest CI length
Less	Non-metastasis	T2-3	0(0/87)	0.00–4.15	8.3
	Non-metastasis	T4a	5.88(2/34)	0.72–19.68	14.4
	Any-metastasis	T2-3	2.17(1/46)	0.00–11.53	11.2
	Any-metastasis	T4a	11.29(7/62)	4.66–21.89	11.2
Gre/Ant/Post/Cir	Non-metastasis	T2-3	0(0/15)	0.00–21.80	16
	Non-metastasis	T4a	16.67(4/24)	4.74–37.38	14.4
	Any-metastasis	T2-3	30(3/10)	6.67–65.25	16
	Any-metastasis	T4a	54.55(18/33)	36.35–71.89	14.4

Gre, Greater curvature; Less, Lesser curvature; Ant, Anterior wall; Post, Posterior wall; Cir, Circum.

## Discussion

As safety and feasibility were comfirmed, since 1994 the advent of lapascopic assisted gastrectomy (LAG) [17], it has been widely accepted. Compared with open surgery, LSPNo.10LND can more easily approach the deep portion and thoroughly dissect No.10 LNs without mobilizing the spleen [10]. Using a middle surgical approach, seven patients underwent this new technique in a single Japanese center, with a mean 2.6±2.8 No. 10 LNs retrieved [18]. In comparison, we retrieved a mean 2.69±2.76 No. 10 LNs, which was considerable with the conventional procedure [4], suggesting that LSPNo.10LND is feasible in patients with advanced proximal gastric cancer.

We found that 35 of 346 patients (10.1%) with proximal gastric cancer and 35 of 311 patients (11.3%) with advanced proximal gastric cancer were positive for SHLNM, in agreement with previous findings [19]–[23]. According to JCGC 3rd edition guideline, for T1 group, there is no need to undergo No.10 LND. In our research, no SHLNM was found in this subgroup (0/35). Moreover, for T1 group, as the incidence of SHLNM was zero, there is no statistical meanings when a comparison made between it and others. So we only involved the T2–T4 groups in the multiple tests. The risk factors for SHLNM remain unclear. An analysis of 219 patients who underwent splenic hilar LN dissection found that the depth of invasion closely correlated with SHLNM. The likelihood of SHLNM in patients with AGC is increased in patients when tumors penetrating the subserosa or the muscularis with more than 7 macroscopic LN metastases [24]. In contrast with tumors located on the other portions, those located on the greater curvature were at higher risk for SHLNM (37.5%, 6/16) [25]. Regional LN metastasis was also related to SHLNM, especially in patients with proximal gastric cancer, with regional LN metastases always accompanied by metastases at No. 1, No. 3, and No.7 [20]. Deeper tumor invasion was associated with a higher risk for SHLNM. The probability of SHLNM was found to depend on primary tumor location. Tumors located in the greater curvature or posterior wall are more likely to spread through the LNs around the LGEVs, SGVs, and posterior gastric vessels (PGVs) to the No.10 LNs. The major lymphatic pathway of SHLNM was from No. 3 LN to No. 7 or No. 11 LN, finally No. 10 LN [26]. Therefore, SHLNM was accompanied mostly by metastases at No. 7 and No. 11 LN, which was consistent with our multiple analysis. Except for other lymph nodes, only the metastases at No. 7 and No. 11 LN were correlated with SHLNM.

Although many studies have assessed the risk factors for SHLNM, none has formulated a predictive model. Multi-variable AUCs were utilized to evaluate the accuracy of the predictive models [27], while AIC was applied to assess the ability of different models to detect SHLNM [28]. By integrating AIC with AUC, we found that the optimal model for proximal gastric cancer included tumor location, depth of invasion, and metastasis status at No. 7 and No. 11 LNs.

Because of low risk for SHLNM, No.10LND was unnecessary for patients with early proximal gastric cancer. Our findings also demonstrate none of them diagnosed with SHLNM (0/35). However, the need for routine performance of NO.10LND in patients with advanced proximal gastric cancer remains unclear. Japanese guidelines recommend NO.10LND for all patients with advanced proximal gastric cancer [2]. One Japanese study, however, suggested that NO.10LND was unnecessary in patients with tumors located on the lesser curvature and without subserosal invasion, since survival rates were similar in patients who did and did not undergo NO.10LND [19]. A prospective study found that no patient with a stage IA, IB, II, or IIIA primary tumor was diagnosed with SHLNM, with NO.10LND not associated with long-term survival and postoperative morbidity rates [23]. Stratification analysis and binary distribution test were applied based on the independent risk factors involved in the optimal predictive model. The lymphatic pathway theory verified that tumors in the lesser curvature primarily invade from No.3 LN to No.7 or No.11 LN and from there to No.10 LN [19], [26], [29]. As extremely low risk for SHLNM, when tumor locates in the lesser curvature without serosa invasion and metastasis to the No.7 and No.11 LN, this subgroup may avoid routine LSPNO.10LND. Whereas all other advanced proximal gastric cancer, with a higher risk of SHLNM, should undergo LSPNO.10LND.

## Supporting Information

Figure S1 **Intraoperative view of laparoscopic spleen-preserving no. 10 lymph node dissection : Anterior view.** Abbreviations: SpA, splenic artery; SpV, splenic vein.
(TIF)

Figure S2 **Intraoperative view of laparoscopic spleen-preserving no. 10 lymph node dissection : Posterior view.** Abbreviations: SpV, splenic vein.
(TIF)
