# Supplementary figures and images for: A 346 Case Analysis for Laparoscopic Spleen-Preserving No.10 Lymph Node Dissection for Proximal Gastric Cancer: A Single Center Study
Source: PLoS One. 2014 Sep 29;9(9):e108480. doi: 10.1371/journal.pone.0108480 (PMC4181621; doi:10.1371/journal.pone.0108480)

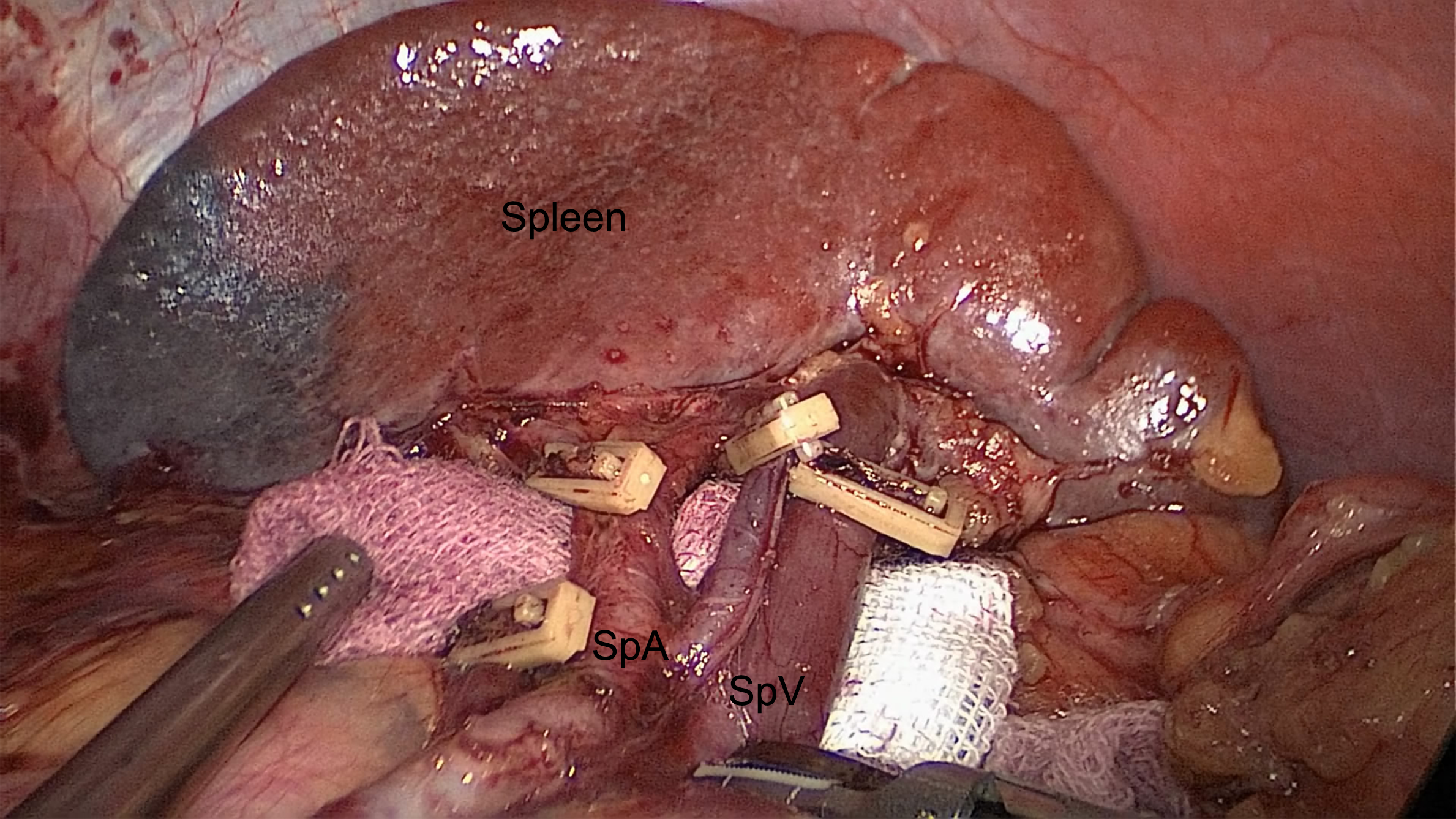

Supplement: Figure S1 — Intraoperative view of laparoscopic spleen-preserving no. 10 lymph node dissection : Anterior view. Abbreviations: SpA, splenic artery; SpV, splenic vein. (TIF) [file pone.0108480.s001.tif]

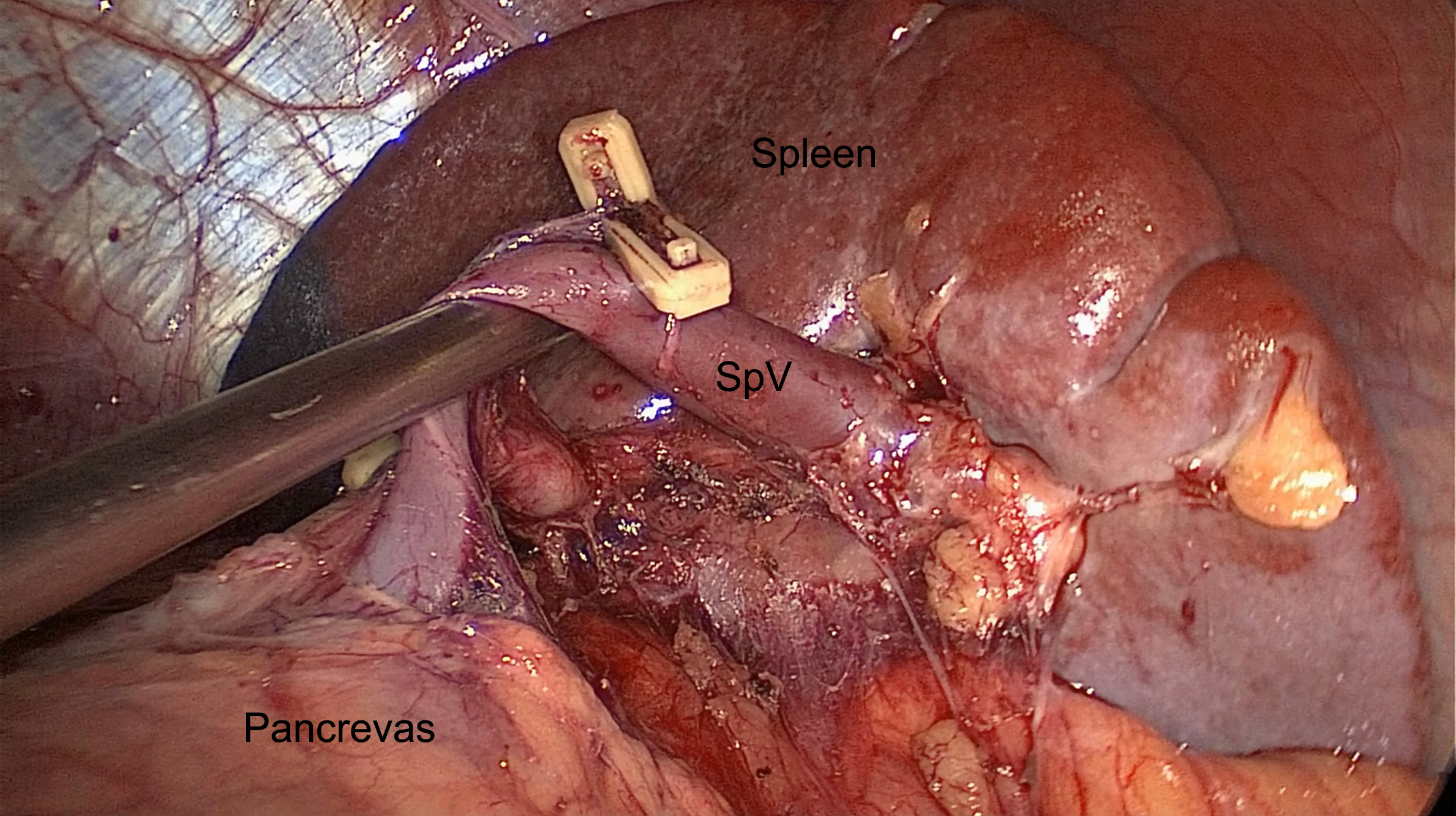

Supplement: Figure S2 — Intraoperative view of laparoscopic spleen-preserving no. 10 lymph node dissection : Posterior view. Abbreviations: SpV, splenic vein. (TIF) [file pone.0108480.s002.tif]
